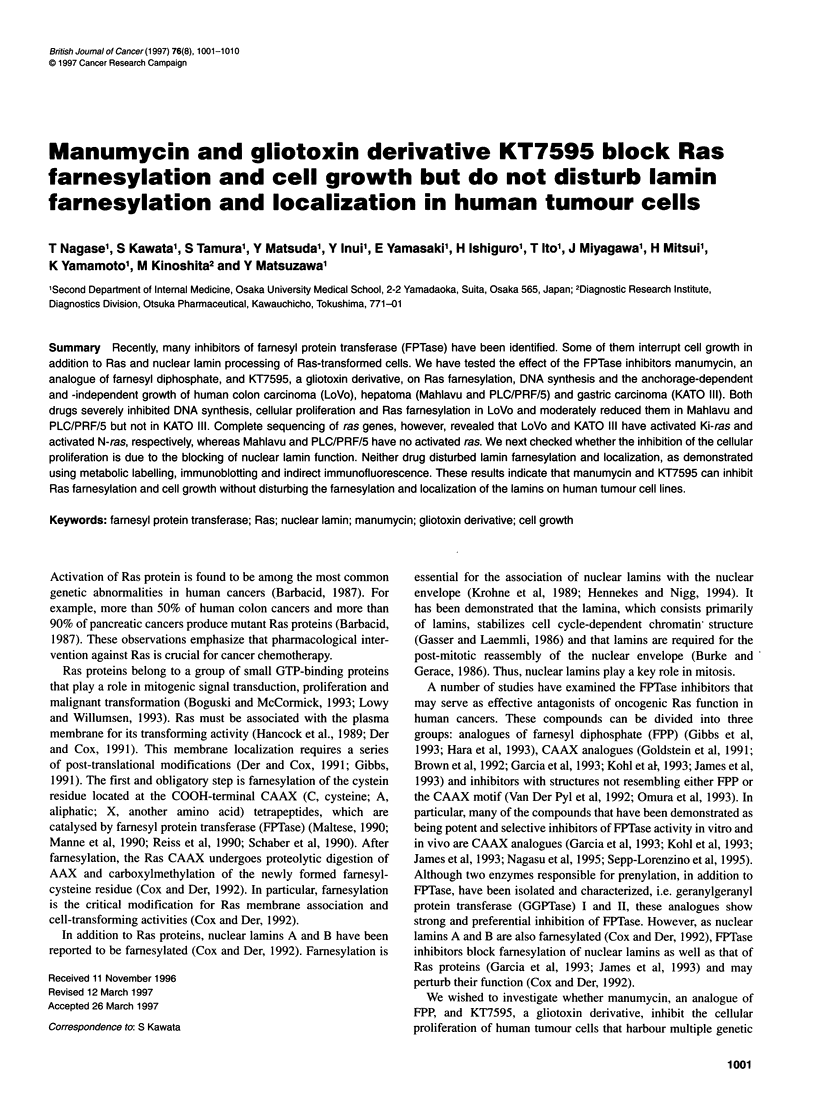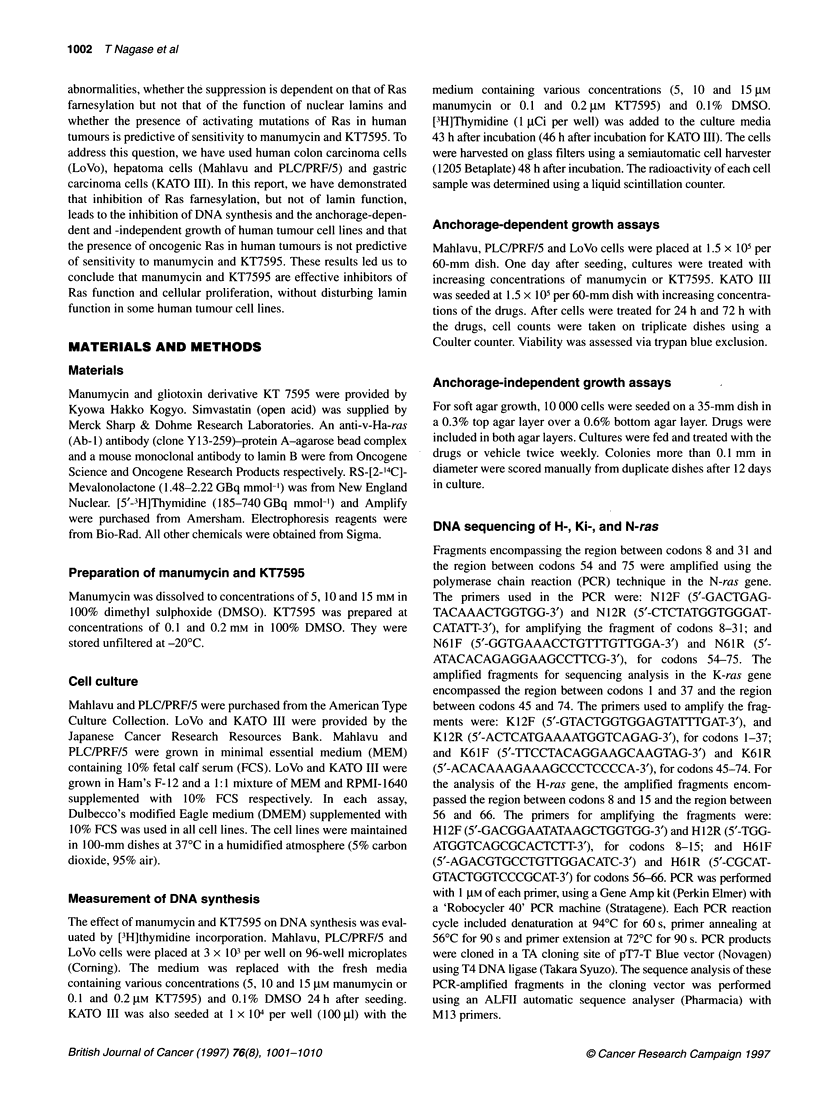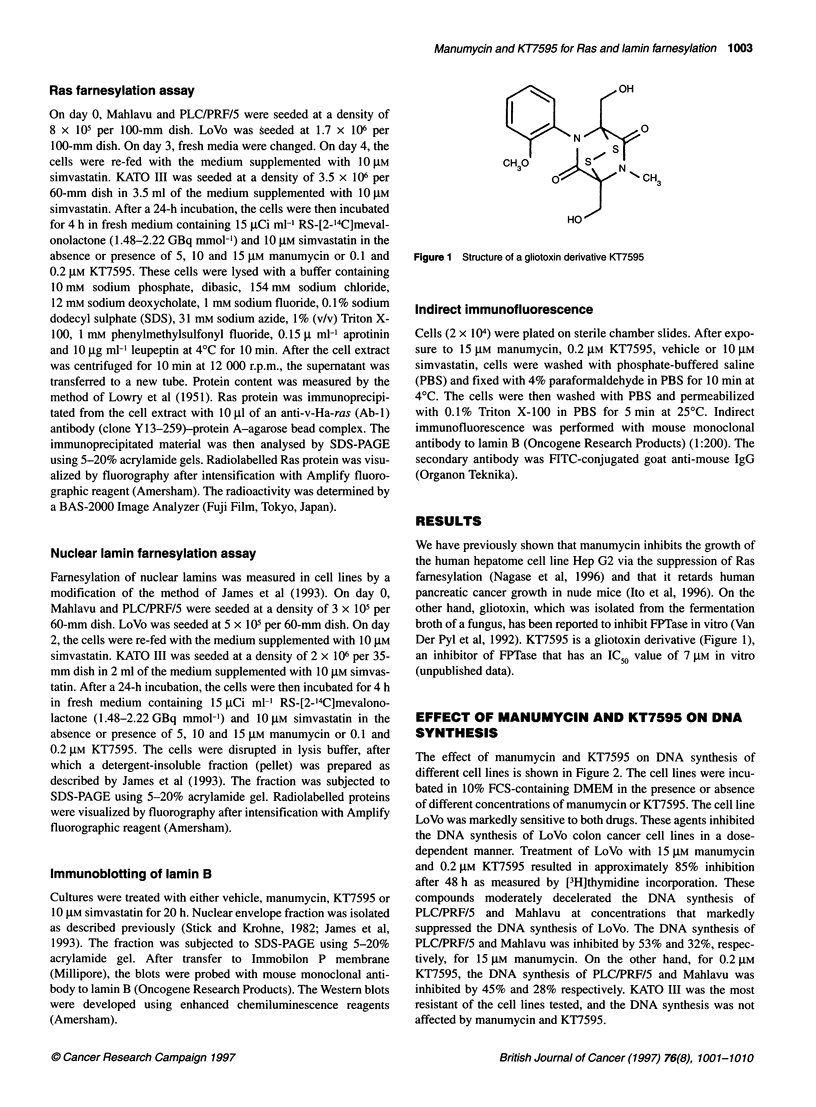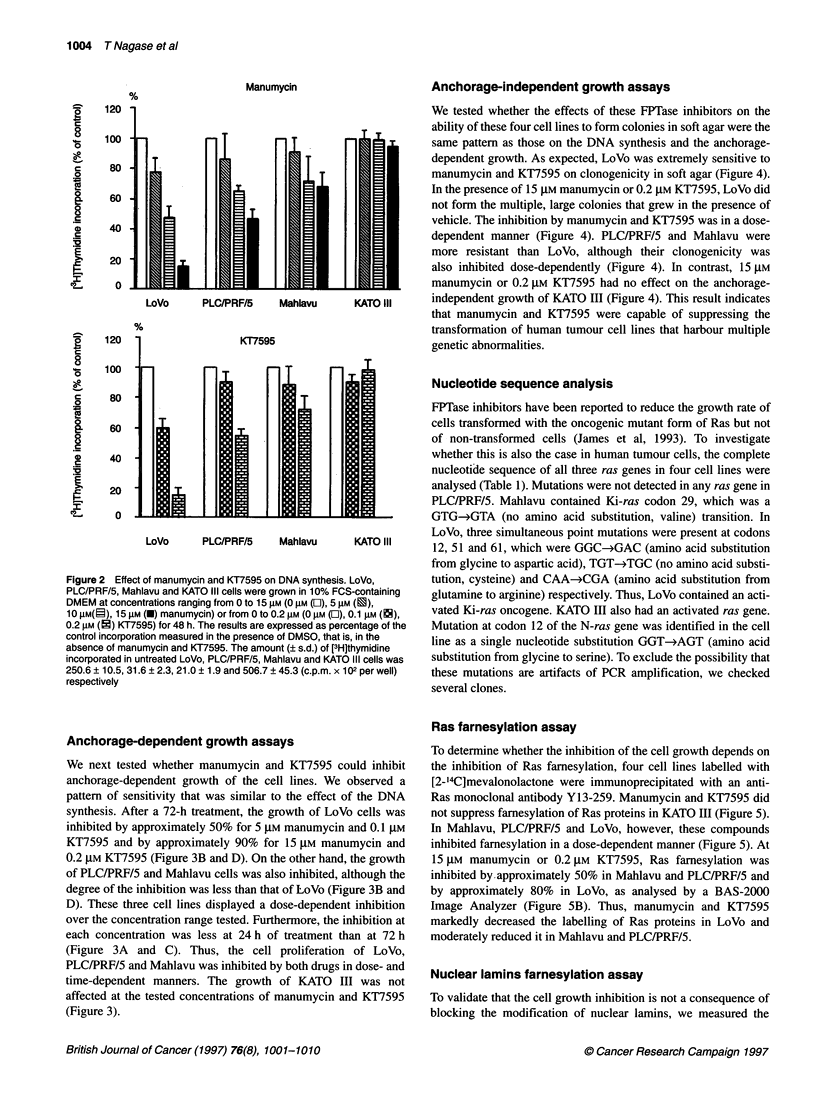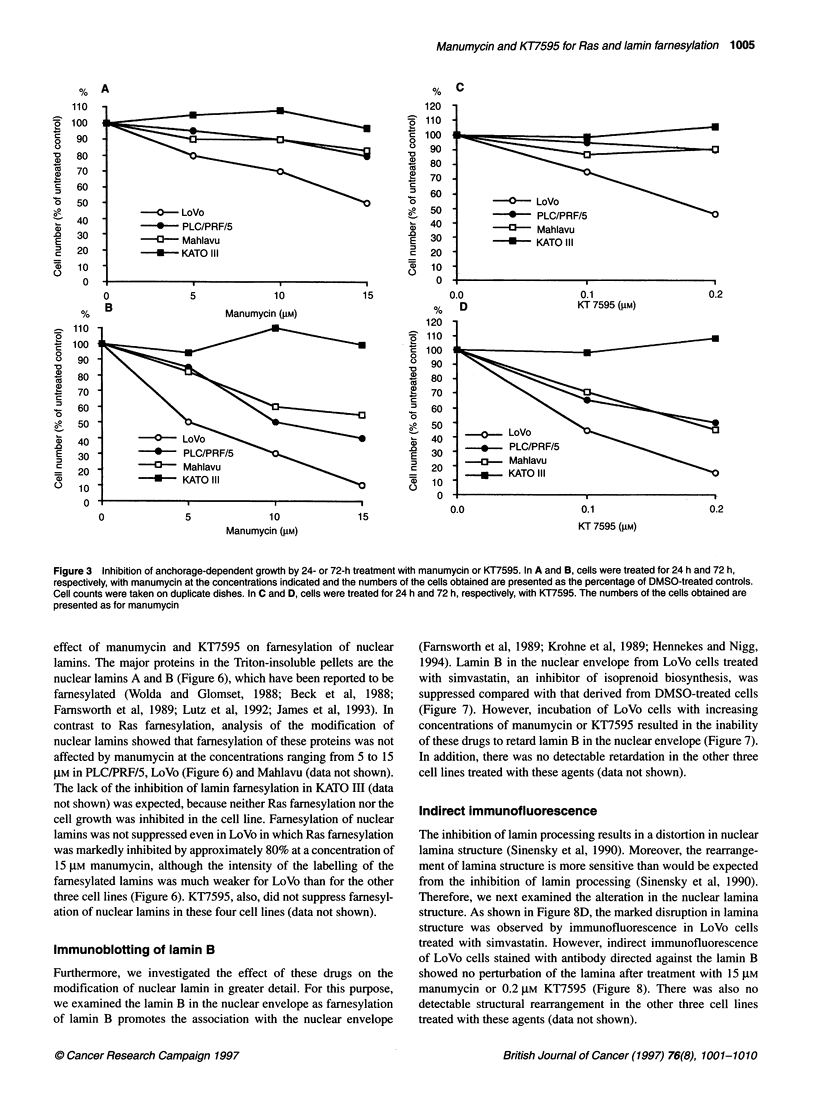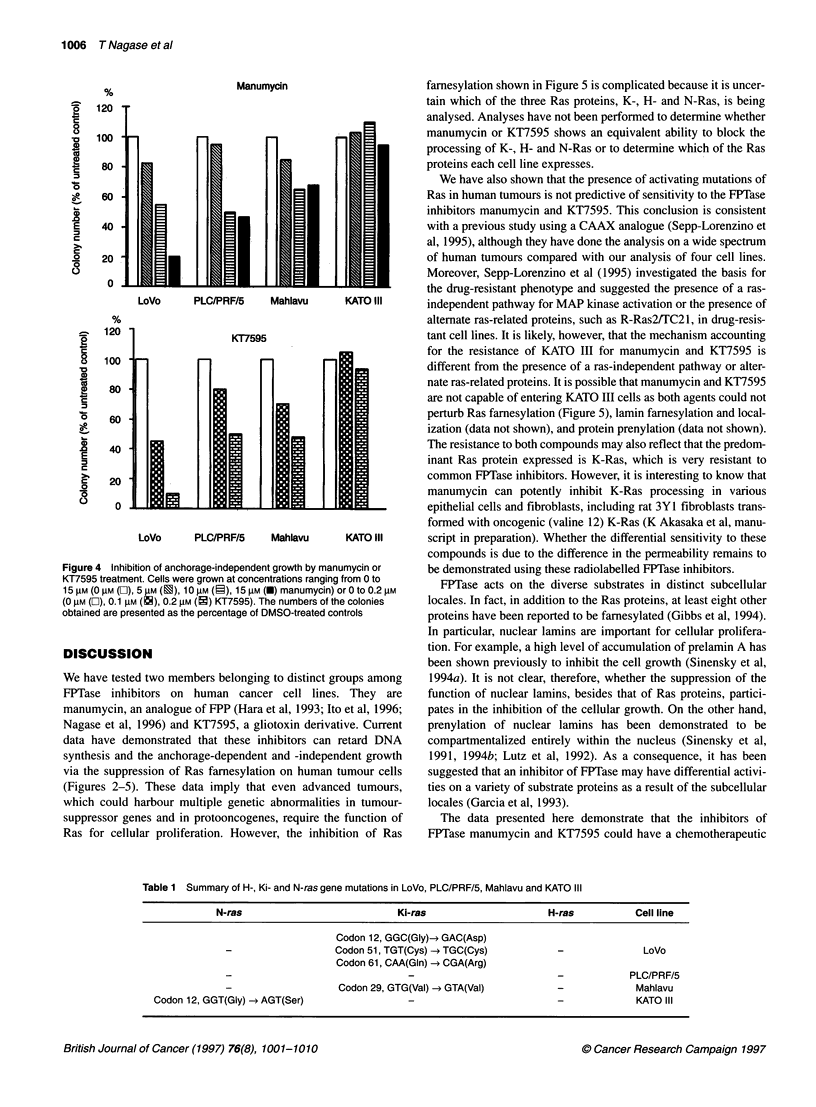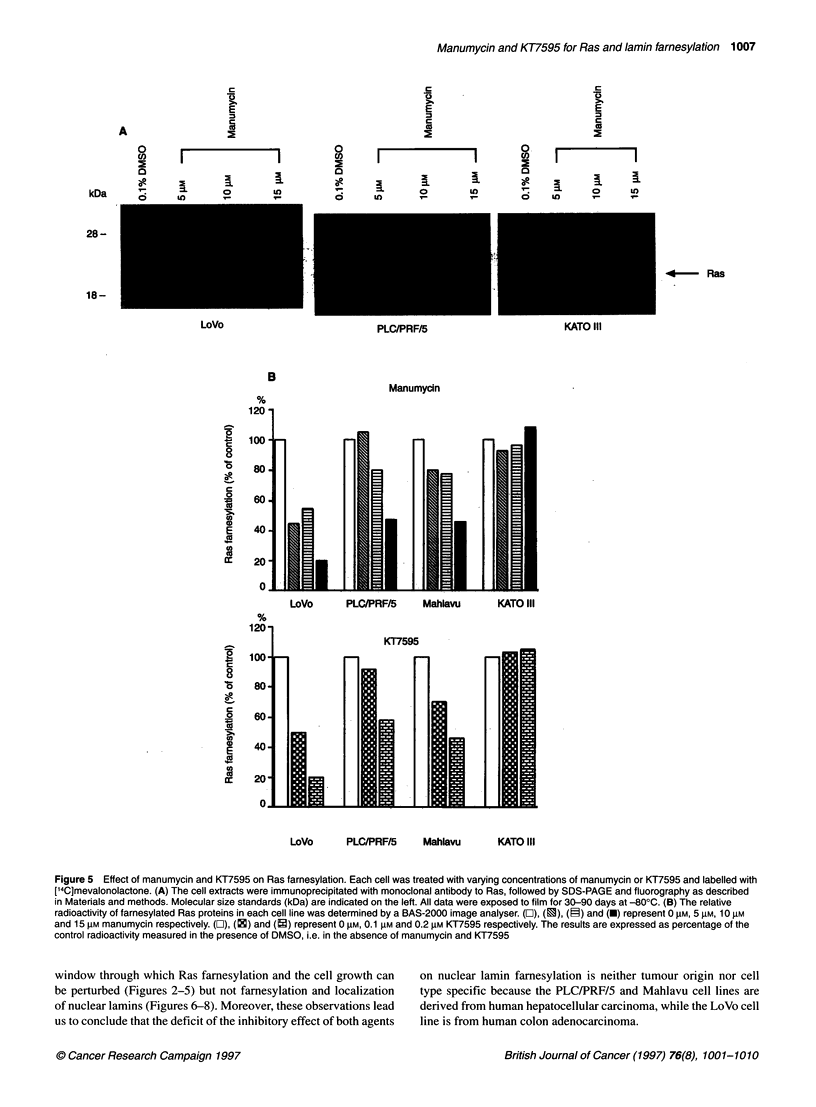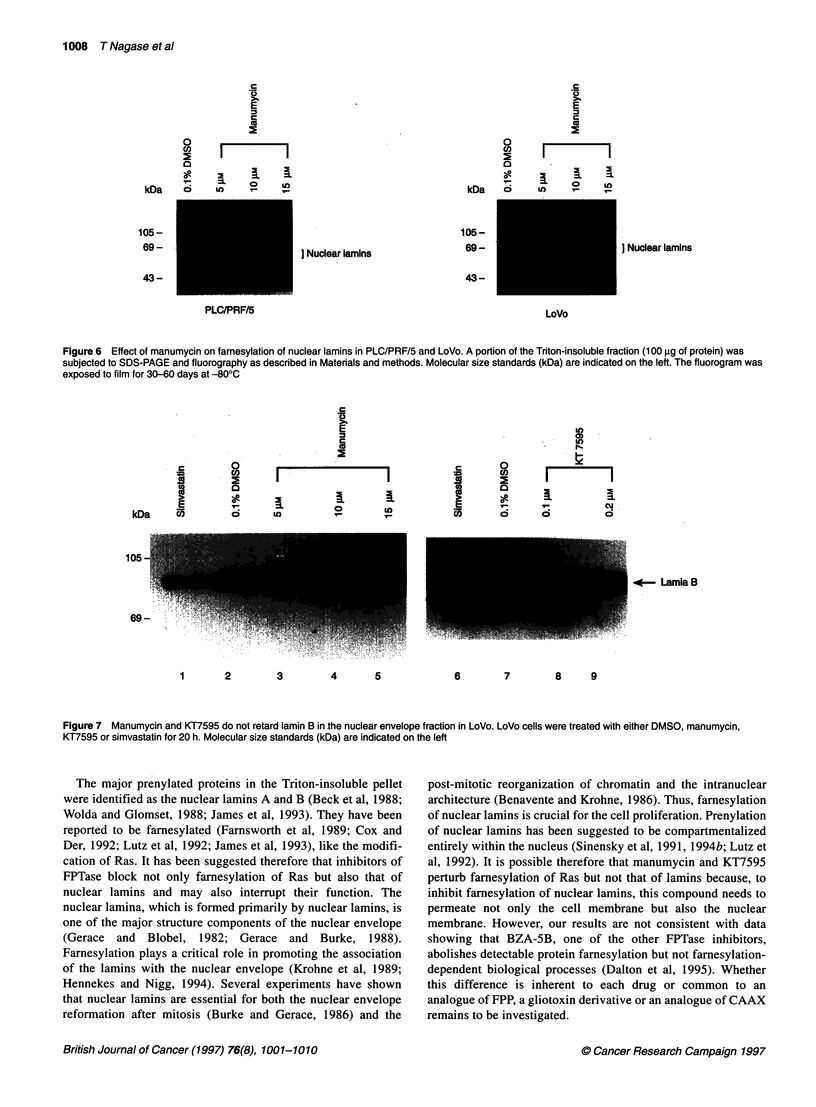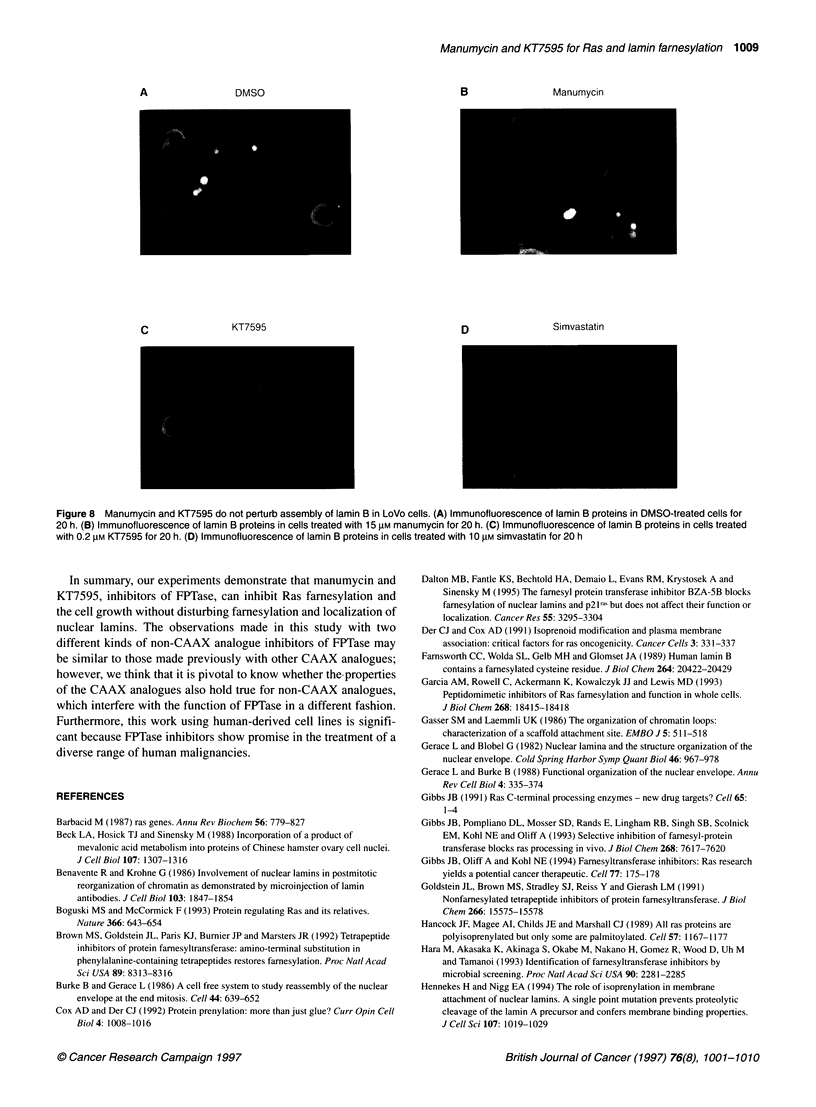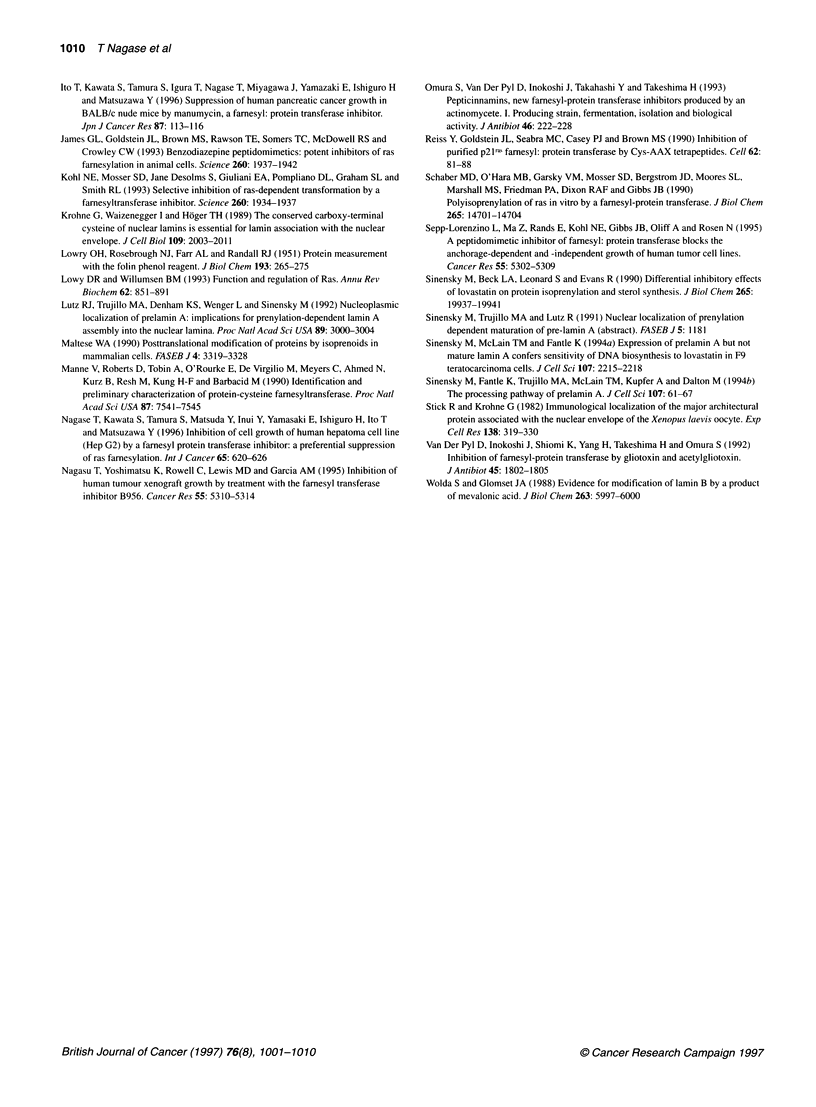# Manumycin and gliotoxin derivative KT7595 block Ras farnesylation and cell growth but do not disturb lamin farnesylation and localization in human tumour cells.

**DOI:** 10.1038/bjc.1997.499

**Published:** 1997

**Authors:** T. Nagase, S. Kawata, S. Tamura, Y. Matsuda, Y. Inui, E. Yamasaki, H. Ishiguro, T. Ito, J. Miyagawa, H. Mitsui, K. Yamamoto, M. Kinoshita, Y. Matsuzawa

**Affiliations:** Second Department of Internal Medicine, Osaka University Medical School, Suita, Japan.

## Abstract

**Images:**


*British Journal of Cancer* (1997) **76**(8), 1001–1010
© 1997 Cancer Research Campaign

### Manumycin and gliotoxin derivative KT7595 block Ras farnesylation and cell growth but do not disturb lamin farnesylation and localization in human tumour cells

**T Nagase[1], S Kawata[1], S Tamura[1], Y Matsuda[1], Y Inui[1], E Yamasaki[1], H Ishiguro[1], T Ito[1], J Miyagawa[1], H Mitsui[1], K Yamamoto[1], M Kinoshita[2] and Y Matsuzawa[1]**

[1]Second Department of Internal Medicine, Osaka University Medical School, 2-2 Yamadaoka, Suita, Osaka 565, Japan; [2]Diagnostic Research Institute, Diagnostics Division, Otsuka Pharmaceutical, Kawauchicho, Tokushima, 771–01

**Summary** Recently, many inhibitors of farnesyl protein transferase (FPTase) have been identified. Some of them interrupt cell growth in addition to Ras and nuclear lamin processing of Ras-transformed cells. We have tested the effect of the FPTase inhibitors manumycin, an analogue of farnesyl diphosphate, and KT7595, a gliotoxin derivative, on Ras farnesylation, DNA synthesis and the anchorage-dependent and -independent growth of human colon carcinoma (LoVo), hepatoma (Mahlavu and PLC/PRF/5) and gastric carcinoma (KATO III). Both drugs severely inhibited DNA synthesis, cellular proliferation and Ras farnesylation in LoVo and moderately reduced them in Mahlavu and PLC/PRF/5 but not in KATO III. Complete sequencing of *ras* genes, however, revealed that LoVo and KATO III have activated Ki-*ras* and activated N-*ras*, respectively, whereas Mahlavu and PLC/PRF/5 have no activated *ras*. We next checked whether the inhibition of the cellular proliferation is due to the blocking of nuclear lamin function. Neither drug disturbed lamin farnesylation and localization, as demonstrated using metabolic labelling, immunoblotting and indirect immunofluorescence. These results indicate that manumycin and KT7595 can inhibit Ras farnesylation and cell growth without disturbing the farnesylation and localization of the lamins on human tumour cell lines.

**Keywords:** farnesyl protein transferase; Ras; nuclear lamin; manumycin; gliotoxin derivative; cell growth

Activation of Ras protein is found to be among the most common genetic abnormalities in human cancers (Barbacid, 1987). For example, more than 50% of human colon cancers and more than 90% of pancreatic cancers produce mutant Ras proteins (Barbacid, 1987). These observations emphasize that pharmacological intervention against Ras is crucial for cancer chemotherapy.

Ras proteins belong to a group of small GTP-binding proteins that play a role in mitogenic signal transduction, proliferation and malignant transformation (Boguski and McCormick, 1993; Lowy and Willumsen, 1993). Ras must be associated with the plasma membrane for its transforming activity (Hancock et al., 1989; Der and Cox, 1991). This membrane localization requires a series of post-translational modifications (Der and Cox, 1991; Gibbs, 1991). The first and obligatory step is farnesylation of the cystein residue located at the COOH-terminal CAAX (C, cysteine; A, aliphatic; X, another amino acid) tetrapeptides, which are catalysed by farnesyl protein transferase (FPTase) (Maltese, 1990; Manne et al, 1990; Reiss et al, 1990; Schaber et al, 1990). After farnesylation, the Ras CAAX undergoes proteolytic digestion of AAX and carboxylmethylation of the newly formed farnesyl-cysteine residue (Cox and Der, 1992). In particular, farnesylation is the critical modification for Ras membrane association and cell-transforming activities (Cox and Der, 1992).

In addition to Ras proteins, nuclear lamins A and B have been reported to be farnesylated (Cox and Der, 1992). Farnesylation is essential for the association of nuclear lamins with the nuclear envelope (Krohne et al, 1989; Hennekes and Nigg, 1994). It has been demonstrated that the lamina, which consists primarily of lamins, stabilizes cell cycle-dependent chromatin structure (Gasser and Laemmli, 1986) and that lamins are required for the post-mitotic reassembly of the nuclear envelope (Burke and Gerace, 1986). Thus, nuclear lamins play a key role in mitosis.

A number of studies have examined the FPTase inhibitors that may serve as effective antagonists of oncogenic Ras function in human cancers. These compounds can be divided into three groups: analogues of farnesyl diphosphate (FPP) (Gibbs et al, 1993; Hara et al, 1993), CAAX analogues (Goldstein et al, 1991; Brown et al, 1992; Garcia et al, 1993; Kohl et al, 1993; James et al, 1993) and inhibitors with structures not resembling either FPP or the CAAX motif (Van Der Pyl et al, 1992; Omura et al, 1993). In particular, many of the compounds that have been demonstrated as being potent and selective inhibitors of FPTase activity in vitro and in vivo are CAAX analogues (Garcia et al, 1993; Kohl et al, 1993; James et al, 1993; Nagasu et al, 1995; Sepp-Lorenzino et al, 1995). Although two enzymes responsible for prenylation, in addition to FPTase, have been isolated and characterized, i.e. geranylgeranyl protein transferase (GGPTase) I and II, these analogues show strong and preferential inhibition of FPTase. However, as nuclear lamins A and B are also farnesylated (Cox and Der, 1992), FPTase inhibitors block farnesylation of nuclear lamins as well as that of Ras proteins (Garcia et al, 1993; James et al, 1993) and may perturb their function (Cox and Der, 1992).

We wished to investigate whether manumycin, an analogue of FPP, and KT7595, a gliotoxin derivative, inhibit the cellular proliferation of human tumour cells that harbour multiple genetic

Received 11 November 1996
Revised 12 March 1997
Accepted 26 March 1997

*Correspondence to:* S Kawata abnormalities, whether the suppression is dependent on that of Ras farnesylation but not that of the function of nuclear lamins and whether the presence of activating mutations of Ras in human tumours is predictive of sensitivity to manumycin and KT7595. To address this question, we have used human colon carcinoma cells (LoVo), hepatoma cells (Mahlavu and PLC/PRF/5) and gastric carcinoma cells (KATO III). In this report, we have demonstrated that inhibition of Ras farnesylation, but not of lamin function, leads to the inhibition of DNA synthesis and the anchorage-dependent and -independent growth of human tumour cell lines and that the presence of oncogenic Ras in human tumours is not predictive of sensitivity to manumycin and KT7595. These results led us to conclude that manumycin and KT7595 are effective inhibitors of Ras function and cellular proliferation, without disturbing lamin function in some human tumour cell lines.

### MATERIALS AND METHODS

### Materials

Manumycin and gliotoxin derivative KT 7595 were provided by Kyowa Hakko Kogyo. Simvastatin (open acid) was supplied by Merck Sharp & Dohme Research Laboratories. An anti-v-Ha-*ras* (Ab-1) antibody (clone Y13-259)–protein A–agarose bead complex and a mouse monoclonal antibody to lamin B were from Oncogene Science and Oncogene Research Products respectively. RS-[2-$^{14}$C]-Mevalonolactone (1.48–2.22 GBq mmol$^{-1}$) was from New England Nuclear. [5′-$^{3}$H]Thymidine (185–740 GBq mmol$^{-1}$) and Amplify were purchased from Amersham. Electrophoresis reagents were from Bio-Rad. All other chemicals were obtained from Sigma.

### Preparation of manumycin and KT7595

Manumycin was dissolved to concentrations of 5, 10 and 15 mM in 100% dimethyl sulphoxide (DMSO). KT7595 was prepared at concentrations of 0.1 and 0.2 mM in 100% DMSO. They were stored unfiltered at –20°C.

### Cell culture

Mahlavu and PLC/PRF/5 were purchased from the American Type Culture Collection. LoVo and KATO III were provided by the Japanese Cancer Research Resources Bank. Mahlavu and PLC/PRF/5 were grown in minimal essential medium (MEM) containing 10% fetal calf serum (FCS). LoVo and KATO III were grown in Ham's F-12 and a 1:1 mixture of MEM and RPMI-1640 supplemented with 10% FCS respectively. In each assay, Dulbecco's modified Eagle medium (DMEM) supplemented with 10% FCS was used in all cell lines. The cell lines were maintained in 100-mm dishes at 37°C in a humidified atmosphere (5% carbon dioxide, 95% air).

### Measurement of DNA synthesis

The effect of manumycin and KT7595 on DNA synthesis was evaluated by [$^{3}$H]thymidine incorporation. Mahlavu, PLC/PRF/5 and LoVo cells were placed at $3 \times 10^3$ per well on 96-well microplates (Corning). The medium was replaced with the fresh media containing various concentrations (5, 10 and 15 μM manumycin or 0.1 and 0.2 μM KT7595) and 0.1% DMSO 24 h after seeding. KATO III was also seeded at $1 \times 10^4$ per well (100 μl) with the medium containing various concentrations (5, 10 and 15 μM manumycin or 0.1 and 0.2 μM KT7595) and 0.1% DMSO. [$^{3}$H]Thymidine (1 μCi per well) was added to the culture media 43 h after incubation (46 h after incubation for KATO III). The cells were harvested on glass filters using a semiautomatic cell harvester (1205 Betaplate) 48 h after incubation. The radioactivity of each cell sample was determined using a liquid scintillation counter.

### Anchorage-dependent growth assays

Mahlavu, PLC/PRF/5 and LoVo cells were placed at $1.5 \times 10^5$ per 60-mm dish. One day after seeding, cultures were treated with increasing concentrations of manumycin or KT7595. KATO III was seeded at $1.5 \times 10^5$ per 60-mm dish with increasing concentrations of the drugs. After cells were treated for 24 h and 72 h with the drugs, cell counts were taken on triplicate dishes using a Coulter counter. Viability was assessed via trypan blue exclusion.

### Anchorage-independent growth assays

For soft agar growth, 10 000 cells were seeded on a 35-mm dish in a 0.3% top agar layer over a 0.6% bottom agar layer. Drugs were included in both agar layers. Cultures were fed and treated with the drugs or vehicle twice weekly. Colonies more than 0.1 mm in diameter were scored manually from duplicate dishes after 12 days in culture.

### DNA sequencing of H-, Ki-, and N-*ras*

Fragments encompassing the region between codons 8 and 31 and the region between codons 54 and 75 were amplified using the polymerase chain reaction (PCR) technique in the N-*ras* gene. The primers used in the PCR were: N12F (5′-GACTGAGTACAAACTGGTGG-3′) and N12R (5′-CTCTATGGTGGGATCATATT-3′), for amplifying the fragment of codons 8–31; and N61F (5′-GGTGAAACCTGTTTGTTGGA-3′) and N61R (5′-ATACACAGAGGAAGCCTTCG-3′), for codons 54–75. The amplified fragments for sequencing analysis in the K-*ras* gene encompassed the region between codons 1 and 37 and the region between codons 45 and 74. The primers used to amplify the fragments were: K12F (5′-GTACTGGTGGAGTATTTGAT-3′), and K12R (5′-ACTCATGAAAATGGTCAGAG-3′), for codons 1–37; and K61F (5′-TTCCTACAGGAAGCAAGTAG-3′) and K61R (5′-ACACAAAGAAAGCCCTCCCCA-3′), for codons 45–74. For the analysis of the H-*ras* gene, the amplified fragments encompassed the region between codons 8 and 15 and the region between 56 and 66. The primers for amplifying the fragments were: H12F (5′-GACGGAATATAAGCTGGTGG-3′) and H12R (5′-TGGATGGTCAGCGCACTCTT-3′), for codons 8–15; and H61F (5′-AGACGTGCCTGTTGGACATC-3′) and H61R (5′-CGCATGTACTGGTCCCGCAT-3′) for codons 56–66. PCR was performed with 1 μM of each primer, using a Gene Amp kit (Perkin Elmer) with a 'Robocycler 40' PCR machine (Stratagene). Each PCR reaction cycle included denaturation at 94°C for 60 s, primer annealing at 56°C for 90 s and primer extension at 72°C for 90 s. PCR products were cloned in a TA cloning site of pT7-T Blue vector (Novagen) using T4 DNA ligase (Takara Syuzo). The sequence analysis of these PCR-amplified fragments in the cloning vector was performed using an ALFII automatic sequence analyser (Pharmacia) with M13 primers.

© Cancer Research Campaign 1997

### Ras farnesylation assay

On day 0, Mahlavu and PLC/PRF/5 were seeded at a density of $8 \times 10^5$ per 100-mm dish. LoVo was seeded at $1.7 \times 10^6$ per 100-mm dish. On day 3, fresh media were changed. On day 4, the cells were re-fed with the medium supplemented with 10 μM simvastatin. KATO III was seeded at a density of $3.5 \times 10^6$ per 60-mm dish in 3.5 ml of the medium supplemented with 10 μM simvastatin. After a 24-h incubation, the cells were then incubated for 4 h in fresh medium containing 15 μCi $ml^{-1}$ RS-[2-$^{14}$C]mevalonolactone (1.48–2.22 GBq $mmol^{-1}$) and 10 μM simvastatin in the absence or presence of 5, 10 and 15 μM manumycin or 0.1 and 0.2 μM KT7595. These cells were lysed with a buffer containing 10 mM sodium phosphate, dibasic, 154 mM sodium chloride, 12 mM sodium deoxycholate, 1 mM sodium fluoride, 0.1% sodium dodecyl sulphate (SDS), 31 mM sodium azide, 1% (v/v) Triton X-100, 1 mM phenylmethylsulfonyl fluoride, 0.15 μ $ml^{-1}$ aprotinin and 10 μg $ml^{-1}$ leupeptin at 4°C for 10 min. After the cell extract was centrifuged for 10 min at 12 000 r.p.m., the supernatant was transferred to a new tube. Protein content was measured by the method of Lowry et al (1951). Ras protein was immunoprecipitated from the cell extract with 10 μl of an anti-v-Ha-*ras* (Ab-1) antibody (clone Y13–259)–protein A–agarose bead complex. The immunoprecipitated material was then analysed by SDS-PAGE using 5–20% acrylamide gels. Radiolabelled Ras protein was visualized by fluorography after intensification with Amplify fluorographic reagent (Amersham). The radioactivity was determined by a BAS-2000 Image Analyzer (Fuji Film, Tokyo, Japan).

### Nuclear lamin farnesylation assay

Farnesylation of nuclear lamins was measured in cell lines by a modification of the method of James et al (1993). On day 0, Mahlavu and PLC/PRF/5 were seeded at a density of $3 \times 10^5$ per 60-mm dish. LoVo was seeded at $5 \times 10^5$ per 60-mm dish. On day 2, the cells were re-fed with the medium supplemented with 10 μM simvastatin. KATO III was seeded at a density of $2 \times 10^6$ per 35-mm dish in 2 ml of the medium supplemented with 10 μM simvastatin. After a 24-h incubation, the cells were then incubated for 4 h in fresh medium containing 15 μCi $ml^{-1}$ RS-[2-$^{14}$C]mevalonolactone (1.48–2.22 GBq $mmol^{-1}$) and 10 μM simvastatin in the absence or presence of 5, 10 and 15 μM manumycin or 0.1 and 0.2 μM KT7595. The cells were disrupted in lysis buffer, after which a detergent-insoluble fraction (pellet) was prepared as described by James et al (1993). The fraction was subjected to SDS-PAGE using 5–20% acrylamide gel. Radiolabelled proteins were visualized by fluorography after intensification with Amplify fluorographic reagent (Amersham).

### Immunoblotting of lamin B

Cultures were treated with either vehicle, manumycin, KT7595 or 10 μM simvastatin for 20 h. Nuclear envelope fraction was isolated as described previously (Stick and Krohne, 1982; James et al, 1993). The fraction was subjected to SDS-PAGE using 5–20% acrylamide gel. After transfer to Immobilon P membrane (Millipore), the blots were probed with mouse monoclonal antibody to lamin B (Oncogene Research Products). The Western blots were developed using enhanced chemiluminescence reagents (Amersham).

**Figure 1** Structure of a gliotoxin derivative KT7595

### Indirect immunofluorescence

Cells ($2 \times 10^4$) were plated on sterile chamber slides. After exposure to 15 μM manumycin, 0.2 μM KT7595, vehicle or 10 μM simvastatin, cells were washed with phosphate-buffered saline (PBS) and fixed with 4% paraformaldehyde in PBS for 10 min at 4°C. The cells were then washed with PBS and permeabilized with 0.1% Triton X-100 in PBS for 5 min at 25°C. Indirect immunofluorescence was performed with mouse monoclonal antibody to lamin B (Oncogene Research Products) (1:200). The secondary antibody was FITC-conjugated goat anti-mouse IgG (Organon Teknika).

### RESULTS

We have previously shown that manumycin inhibits the growth of the human hepatome cell line Hep G2 via the suppression of Ras farnesylation (Nagase et al, 1996) and that it retards human pancreatic cancer growth in nude mice (Ito et al, 1996). On the other hand, gliotoxin, which was isolated from the fermentation broth of a fungus, has been reported to inhibit FPTase in vitro (Van Der Pyl et al, 1992). KT7595 is a gliotoxin derivative (Figure 1), an inhibitor of FPTase that has an $IC_{50}$ value of 7 μM in vitro (unpublished data).

### EFFECT OF MANUMYCIN AND KT7595 ON DNA SYNTHESIS

The effect of manumycin and KT7595 on DNA synthesis of different cell lines is shown in Figure 2. The cell lines were incubated in 10% FCS-containing DMEM in the presence or absence of different concentrations of manumycin or KT7595. The cell line LoVo was markedly sensitive to both drugs. These agents inhibited the DNA synthesis of LoVo colon cancer cell lines in a dose-dependent manner. Treatment of LoVo with 15 μM manumycin and 0.2 μM KT7595 resulted in approximately 85% inhibition after 48 h as measured by [$^3$H]thymidine incorporation. These compounds moderately decelerated the DNA synthesis of PLC/PRF/5 and Mahlavu at concentrations that markedly suppressed the DNA synthesis of LoVo. The DNA synthesis of PLC/PRF/5 and Mahlavu was inhibited by 53% and 32%, respectively, for 15 μM manumycin. On the other hand, for 0.2 μM KT7595, the DNA synthesis of PLC/PRF/5 and Mahlavu was inhibited by 45% and 28% respectively. KATO III was the most resistant of the cell lines tested, and the DNA synthesis was not affected by manumycin and KT7595.

© Cancer Research Campaign 1997

**Figure 2** Effect of manumycin and KT7595 on DNA synthesis. LoVo, PLC/PRF/5, Mahlavu and KATO III cells were grown in 10% FCS-containing DMEM at concentrations ranging from 0 to 15 μM (0 μM (□), 5 μM (▧), 10 μM(▤), 15 μM (■) manumycin) or from 0 to 0.2 μM (0 μM (□), 0.1 μM (▩), 0.2 μM (▦) KT7595) for 48 h. The results are expressed as percentage of the control incorporation measured in the presence of DMSO, that is, in the absence of manumycin and KT7595. The amount (± s.d.) of [$^3$H]thymidine incorporated in untreated LoVo, PLC/PRF/5, Mahlavu and KATO III cells was 250.6 ± 10.5, 31.6 ± 2.3, 21.0 ± 1.9 and 506.7 ± 45.3 (c.p.m. × $10^2$ per well) respectively

### Anchorage-dependent growth assays

We next tested whether manumycin and KT7595 could inhibit anchorage-dependent growth of the cell lines. We observed a pattern of sensitivity that was similar to the effect of the DNA synthesis. After a 72-h treatment, the growth of LoVo cells was inhibited by approximately 50% for 5 μM manumycin and 0.1 μM KT7595 and by approximately 90% for 15 μM manumycin and 0.2 μM KT7595 (Figure 3B and D). On the other hand, the growth of PLC/PRF/5 and Mahlavu cells was also inhibited, although the degree of the inhibition was less than that of LoVo (Figure 3B and D). These three cell lines displayed a dose-dependent inhibition over the concentration range tested. Furthermore, the inhibition at each concentration was less at 24 h of treatment than at 72 h (Figure 3A and C). Thus, the cell proliferation of LoVo, PLC/PRF/5 and Mahlavu was inhibited by both drugs in dose- and time-dependent manners. The growth of KATO III was not affected at the tested concentrations of manumycin and KT7595 (Figure 3).

### Anchorage-independent growth assays

We tested whether the effects of these FPTase inhibitors on the ability of these four cell lines to form colonies in soft agar were the same pattern as those on the DNA synthesis and the anchorage-dependent growth. As expected, LoVo was extremely sensitive to manumycin and KT7595 on clonogenicity in soft agar (Figure 4). In the presence of 15 μM manumycin or 0.2 μM KT7595, LoVo did not form the multiple, large colonies that grew in the presence of vehicle. The inhibition by manumycin and KT7595 was in a dose-dependent manner (Figure 4). PLC/PRF/5 and Mahlavu were more resistant than LoVo, although their clonogenicity was also inhibited dose-dependently (Figure 4). In contrast, 15 μM manumycin or 0.2 μM KT7595 had no effect on the anchorage-independent growth of KATO III (Figure 4). This result indicates that manumycin and KT7595 were capable of suppressing the transformation of human tumour cell lines that harbour multiple genetic abnormalities.

### Nucleotide sequence analysis

FPTase inhibitors have been reported to reduce the growth rate of cells transformed with the oncogenic mutant form of Ras but not of non-transformed cells (James et al, 1993). To investigate whether this is also the case in human tumour cells, the complete nucleotide sequence of all three *ras* genes in four cell lines were analysed (Table 1). Mutations were not detected in any *ras* gene in PLC/PRF/5. Mahlavu contained Ki-*ras* codon 29, which was a GTG→GTA (no amino acid substitution, valine) transition. In LoVo, three simultaneous point mutations were present at codons 12, 51 and 61, which were GGC→GAC (amino acid substitution from glycine to aspartic acid), TGT→TGC (no amino acid substitution, cysteine) and CAA→CGA (amino acid substitution from glutamine to arginine) respectively. Thus, LoVo contained an activated Ki-*ras* oncogene. KATO III also had an activated *ras* gene. Mutation at codon 12 of the N-*ras* gene was identified in the cell line as a single nucleotide substitution GGT→AGT (amino acid substitution from glycine to serine). To exclude the possibility that these mutations are artifacts of PCR amplification, we checked several clones.

### Ras farnesylation assay

To determine whether the inhibition of the cell growth depends on the inhibition of Ras farnesylation, four cell lines labelled with [2-$^{14}$C]mevalonolactone were immunoprecipitated with an anti-Ras monoclonal antibody Y13-259. Manumycin and KT7595 did not suppress farnesylation of Ras proteins in KATO III (Figure 5). In Mahlavu, PLC/PRF/5 and LoVo, however, these compounds inhibited farnesylation in a dose-dependent manner (Figure 5). At 15 μM manumycin or 0.2 μM KT7595, Ras farnesylation was inhibited by approximately 50% in Mahlavu and PLC/PRF/5 and by approximately 80% in LoVo, as analysed by a BAS-2000 Image Analyzer (Figure 5B). Thus, manumycin and KT7595 markedly decreased the labelling of Ras proteins in LoVo and moderately reduced it in Mahlavu and PLC/PRF/5.

### Nuclear lamins farnesylation assay

To validate that the cell growth inhibition is not a consequence of blocking the modification of nuclear lamins, we measured the

*© Cancer Research Campaign 1997*

**Figure 3** Inhibition of anchorage-dependent growth by 24- or 72-h treatment with manumycin or KT7595. In **A** and **B**, cells were treated for 24 h and 72 h, respectively, with manumycin at the concentrations indicated and the numbers of the cells obtained are presented as the percentage of DMSO-treated controls. Cell counts were taken on duplicate dishes. In **C** and **D**, cells were treated for 24 h and 72 h, respectively, with KT7595. The numbers of the cells obtained are presented as for manumycin effect of manumycin and KT7595 on farnesylation of nuclear lamins. The major proteins in the Triton-insoluble pellets are the nuclear lamins A and B (Figure 6), which have been reported to be farnesylated (Wolda and Glomset, 1988; Beck et al, 1988; Farnsworth et al, 1989; Lutz et al, 1992; James et al, 1993). In contrast to Ras farnesylation, analysis of the modification of nuclear lamins showed that farnesylation of these proteins was not affected by manumycin at the concentrations ranging from 5 to 15 μM in PLC/PRF/5, LoVo (Figure 6) and Mahlavu (data not shown). The lack of the inhibition of lamin farnesylation in KATO III (data not shown) was expected, because neither Ras farnesylation nor the cell growth was inhibited in the cell line. Farnesylation of nuclear lamins was not suppressed even in LoVo in which Ras farnesylation was markedly inhibited by approximately 80% at a concentration of 15 μM manumycin, although the intensity of the labelling of the farnesylated lamins was much weaker for LoVo than for the other three cell lines (Figure 6). KT7595, also, did not suppress farnesylation of nuclear lamins in these four cell lines (data not shown).

### Immunoblotting of lamin B

Furthermore, we investigated the effect of these drugs on the modification of nuclear lamin in greater detail. For this purpose, we examined the lamin B in the nuclear envelope as farnesylation of lamin B promotes the association with the nuclear envelope (Farnsworth et al, 1989; Krohne et al, 1989; Hennekes and Nigg, 1994). Lamin B in the nuclear envelope from LoVo cells treated with simvastatin, an inhibitor of isoprenoid biosynthesis, was suppressed compared with that derived from DMSO-treated cells (Figure 7). However, incubation of LoVo cells with increasing concentrations of manumycin or KT7595 resulted in the inability of these drugs to retard lamin B in the nuclear envelope (Figure 7). In addition, there was no detectable retardation in the other three cell lines treated with these agents (data not shown).

### Indirect immunofluorescence

The inhibition of lamin processing results in a distortion in nuclear lamina structure (Sinensky et al, 1990). Moreover, the rearrangement of lamina structure is more sensitive than would be expected from the inhibition of lamin processing (Sinensky et al, 1990). Therefore, we next examined the alteration in the nuclear lamina structure. As shown in Figure 8D, the marked disruption in lamina structure was observed by immunofluorescence in LoVo cells treated with simvastatin. However, indirect immunofluorescence of LoVo cells stained with antibody directed against the lamin B showed no perturbation of the lamina after treatment with 15 μM manumycin or 0.2 μM KT7595 (Figure 8). There was also no detectable structural rearrangement in the other three cell lines treated with these agents (data not shown).

© Cancer Research Campaign 1997

**Figure 4** Inhibition of anchorage-independent growth by manumycin or KT7595 treatment. Cells were grown at concentrations ranging from 0 to 15 μM (0 μM (□), 5 μM (▧), 10 μM (▤), 15 μM (■) manumycin) or 0 to 0.2 μM (0 μM (□), 0.1 μM (▩), 0.2 μM (▦) KT7595). The numbers of the colonies obtained are presented as the percentage of DMSO-treated controls

### DISCUSSION

We have tested two members belonging to distinct groups among FPTase inhibitors on human cancer cell lines. They are manumycin, an analogue of FPP (Hara et al, 1993; Ito et al, 1996; Nagase et al, 1996) and KT7595, a gliotoxin derivative. Current data have demonstrated that these inhibitors can retard DNA synthesis and the anchorage-dependent and -independent growth via the suppression of Ras farnesylation on human tumour cells (Figures 2–5). These data imply that even advanced tumours, which could harbour multiple genetic abnormalities in tumour-suppressor genes and in protooncogenes, require the function of Ras for cellular proliferation. However, the inhibition of Ras farnesylation shown in Figure 5 is complicated because it is uncertain which of the three Ras proteins, K-, H- and N-Ras, is being analysed. Analyses have not been performed to determine whether manumycin or KT7595 shows an equivalent ability to block the processing of K-, H- and N-Ras or to determine which of the Ras proteins each cell line expresses.

We have also shown that the presence of activating mutations of Ras in human tumours is not predictive of sensitivity to the FPTase inhibitors manumycin and KT7595. This conclusion is consistent with a previous study using a CAAX analogue (Sepp-Lorenzino et al, 1995), although they have done the analysis on a wide spectrum of human tumours compared with our analysis of four cell lines. Moreover, Sepp-Lorenzino et al (1995) investigated the basis for the drug-resistant phenotype and suggested the presence of a ras-independent pathway for MAP kinase activation or the presence of alternate ras-related proteins, such as R-Ras2/TC21, in drug-resistant cell lines. It is likely, however, that the mechanism accounting for the resistance of KATO III for manumycin and KT7595 is different from the presence of a ras-independent pathway or alternate ras-related proteins. It is possible that manumycin and KT7595 are not capable of entering KATO III cells as both agents could not perturb Ras farnesylation (Figure 5), lamin farnesylation and localization (data not shown), and protein prenylation (data not shown). The resistance to both compounds may also reflect that the predominant Ras protein expressed is K-Ras, which is very resistant to common FPTase inhibitors. However, it is interesting to know that manumycin can potently inhibit K-Ras processing in various epithelial cells and fibroblasts, including rat 3Y1 fibroblasts transformed with oncogenic (valine 12) K-Ras (K Akasaka et al, manuscript in preparation). Whether the differential sensitivity to these compounds is due to the difference in the permeability remains to be demonstrated using these radiolabelled FPTase inhibitors.

FPTase acts on the diverse substrates in distinct subcellular locales. In fact, in addition to the Ras proteins, at least eight other proteins have been reported to be farnesylated (Gibbs et al, 1994). In particular, nuclear lamins are important for cellular proliferation. For example, a high level of accumulation of prelamin A has been shown previously to inhibit the cell growth (Sinensky et al, 1994*a*). It is not clear, therefore, whether the suppression of the function of nuclear lamins, besides that of Ras proteins, participates in the inhibition of the cellular growth. On the other hand, prenylation of nuclear lamins has been demonstrated to be compartmentalized entirely within the nucleus (Sinensky et al, 1991, 1994*b*; Lutz et al, 1992). As a consequence, it has been suggested that an inhibitor of FPTase may have differential activities on a variety of substrate proteins as a result of the subcellular locales (Garcia et al, 1993).

The data presented here demonstrate that the inhibitors of FPTase manumycin and KT7595 could have a chemotherapeutic

**Table 1** Summary of H-, Ki- and N-*ras* gene mutations in LoVo, PLC/PRF/5, Mahlavu and KATO III

| N-*ras* | Ki-*ras* | H-*ras* | Cell line |
|---|---|---|---|
| – | Codon 12, GGC(Gly)→ GAC(Asp)<br>Codon 51, TGT(Cys) → TGC(Cys)<br>Codon 61, CAA(Gln) → CGA(Arg) | – | LoVo |
| – | – | – | PLC/PRF/5 |
| – | Codon 29, GTG(Val) → GTA(Val) | – | Mahlavu |
| Codon 12, GGT(Gly) → AGT(Ser) | – | – | KATO III |

*© Cancer Research Campaign 1997*

**Figure 5** Effect of manumycin and KT7595 on Ras farnesylation. Each cell was treated with varying concentrations of manumycin or KT7595 and labelled with [$^{14}$C]mevalonolactone. (**A**) The cell extracts were immunoprecipitated with monoclonal antibody to Ras, followed by SDS-PAGE and fluorography as described in Materials and methods. Molecular size standards (kDa) are indicated on the left. All data were exposed to film for 30–90 days at –80°C. (**B**) The relative radioactivity of farnesylated Ras proteins in each cell line was determined by a BAS-2000 image analyser. (□), (▧), (⊟) and (■) represent 0 μM, 5 μM, 10 μM and 15 μM manumycin respectively. (□), (▩) and (⊞) represent 0 μM, 0.1 μM and 0.2 μM KT7595 respectively. The results are expressed as percentage of the control radioactivity measured in the presence of DMSO, i.e. in the absence of manumycin and KT7595

window through which Ras farnesylation and the cell growth can be perturbed (Figures 2–5) but not farnesylation and localization of nuclear lamins (Figures 6–8). Moreover, these observations lead us to conclude that the deficit of the inhibitory effect of both agents on nuclear lamin farnesylation is neither tumour origin nor cell type specific because the PLC/PRF/5 and Mahlavu cell lines are derived from human hepatocellular carcinoma, while the LoVo cell line is from human colon adenocarcinoma.

*© Cancer Research Campaign 1997*

**Figure 6** Effect of manumycin on farnesylation of nuclear lamins in PLC/PRF/5 and LoVo. A portion of the Triton-insoluble fraction (100 μg of protein) was subjected to SDS-PAGE and fluorography as described in Materials and methods. Molecular size standards (kDa) are indicated on the left. The fluorogram was exposed to film for 30–60 days at –80°C

**Figure 7** Manumycin and KT7595 do not retard lamin B in the nuclear envelope fraction in LoVo. LoVo cells were treated with either DMSO, manumycin, KT7595 or simvastatin for 20 h. Molecular size standards (kDa) are indicated on the left

The major prenylated proteins in the Triton-insoluble pellet were identified as the nuclear lamins A and B (Beck et al, 1988; Wolda and Glomset, 1988; James et al, 1993). They have been reported to be farnesylated (Farnsworth et al, 1989; Cox and Der, 1992; Lutz et al, 1992; James et al, 1993), like the modification of Ras. It has been suggested therefore that inhibitors of FPTase block not only farnesylation of Ras but also that of nuclear lamins and may also interrupt their function. The nuclear lamina, which is formed primarily by nuclear lamins, is one of the major structure components of the nuclear envelope (Gerace and Blobel, 1982; Gerace and Burke, 1988). Farnesylation plays a critical role in promoting the association of the lamins with the nuclear envelope (Krohne et al, 1989; Hennekes and Nigg, 1994). Several experiments have shown that nuclear lamins are essential for both the nuclear envelope reformation after mitosis (Burke and Gerace, 1986) and the post-mitotic reorganization of chromatin and the intranuclear architecture (Benavente and Krohne, 1986). Thus, farnesylation of nuclear lamins is crucial for the cell proliferation. Prenylation of nuclear lamins has been suggested to be compartmentalized entirely within the nucleus (Sinensky et al, 1991, 1994*b*; Lutz et al, 1992). It is possible therefore that manumycin and KT7595 perturb farnesylation of Ras but not that of lamins because, to inhibit farnesylation of nuclear lamins, this compound needs to permeate not only the cell membrane but also the nuclear membrane. However, our results are not consistent with data showing that BZA-5B, one of the other FPTase inhibitors, abolishes detectable protein farnesylation but not farnesylation-dependent biological processes (Dalton et al, 1995). Whether this difference is inherent to each drug or common to an analogue of FPP, a gliotoxin derivative or an analogue of CAAX remains to be investigated.

 © Cancer Research Campaign 1997

**Figure 8** Manumycin and KT7595 do not perturb assembly of lamin B in LoVo cells. (**A**) Immunofluorescence of lamin B proteins in DMSO-treated cells for 20 h. (**B**) Immunofluorescence of lamin B proteins in cells treated with 15 μM manumycin for 20 h. (**C**) Immunofluorescence of lamin B proteins in cells treated with 0.2 μM KT7595 for 20 h. (**D**) Immunofluorescence of lamin B proteins in cells treated with 10 μM simvastatin for 20 h

In summary, our experiments demonstrate that manumycin and KT7595, inhibitors of FPTase, can inhibit Ras farnesylation and the cell growth without disturbing farnesylation and localization of nuclear lamins. The observations made in this study with two different kinds of non-CAAX analogue inhibitors of FPTase may be similar to those made previously with other CAAX analogues; however, we think that it is pivotal to know whether the properties of the CAAX analogues also hold true for non-CAAX analogues, which interfere with the function of FPTase in a different fashion. Furthermore, this work using human-derived cell lines is significant because FPTase inhibitors show promise in the treatment of a diverse range of human malignancies.

### REFERENCES

Barbacid M (1987) ras genes. *Annu Rev Biochem* **56**: 779–827

Beck LA, Hosick TJ and Sinensky M (1988) Incorporation of a product of mevalonic acid metabolism into proteins of Chinese hamster ovary cell nuclei. *J Cell Biol* **107**: 1307–1316

Benavente R and Krohne G (1986) Involvement of nuclear lamins in postmitotic reorganization of chromatin as demonstrated by microinjection of lamin antibodies. *J Cell Biol* **103**: 1847–1854

Boguski MS and McCormick F (1993) Protein regulating Ras and its relatives. *Nature* **366**: 643–654

Brown MS, Goldstein JL, Paris KJ, Burnier JP and Marsters JR (1992) Tetrapeptide inhibitors of protein farnesyltransferase: amino-terminal substitution in phenylalanine-containing tetrapeptides restores farnesylation. *Proc Natl Acad Sci USA* **89**: 8313–8316

Burke B and Gerace L (1986) A cell free system to study reassembly of the nuclear envelope at the end mitosis. *Cell* **44**: 639–652

Cox AD and Der CJ (1992) Protein prenylation: more than just glue? *Curr Opin Cell Biol* **4**: 1008–1016

Dalton MB, Fantle KS, Bechtold HA, Demaio L, Evans RM, Krystosek A and Sinensky M (1995) The farnesyl protein transferase inhibitor BZA-5B blocks farnesylation of nuclear lamins and p21[ras] but does not affect their function or localization. *Cancer Res* **55**: 3295–3304

Der CJ and Cox AD (1991) Isoprenoid modification and plasma membrane association: critical factors for ras oncogenicity. *Cancer Cells* **3**: 331–337

Farnsworth CC, Wolda SL, Gelb MH and Glomset JA (1989) Human lamin B contains a farnesylated cysteine residue. *J Biol Chem* **264**: 20422–20429

Garcia AM, Rowell C, Ackermann K, Kowalczyk JJ and Lewis MD (1993) Peptidomimetic inhibitors of Ras farnesylation and function in whole cells. *J Biol Chem* **268**: 18415–18418

Gasser SM and Laemmli UK (1986) The organization of chromatin loops: characterization of a scaffold attachment site. *EMBO J* **5**: 511–518

Gerace L and Blobel G (1982) Nuclear lamina and the structure organization of the nuclear envelope. *Cold Spring Harbor Symp Quant Biol* **46**: 967–978

Gerace L and Burke B (1988) Functional organization of the nuclear envelope. *Annu Rev Cell Biol* **4**: 335–374

Gibbs JB (1991) Ras C-terminal processing enzymes – new drug targets? *Cell* **65**: 1–4

Gibbs JB, Pompliano DL, Mosser SD, Rands E, Lingham RB, Singh SB, Scolnick EM, Kohl NE and Oliff A (1993) Selective inhibition of farnesyl-protein transferase blocks ras processing in vivo. *J Biol Chem* **268**: 7617–7620

Gibbs JB, Oliff A and Kohl NE (1994) Farnesyltransferase inhibitors: Ras research yields a potential cancer therapeutic. *Cell* **77**: 175–178

Goldstein JL, Brown MS, Stradley SJ, Reiss Y and Gierash LM (1991) Nonfarnesylated tetrapeptide inhibitors of protein farnesyltransferase. *J Biol Chem* **266**: 15575–15578

Hancock JF, Magee AI, Childs JE and Marshall CJ (1989) All ras proteins are polyisoprenylated but only some are palmitoylated. *Cell* **57**: 1167–1177

Hara M, Akasaka K, Akinaga S, Okabe M, Nakano H, Gomez R, Wood D, Uh M and Tamanoi (1993) Identification of farnesyltransferase inhibitors by microbial screening. *Proc Natl Acad Sci USA* **90**: 2281–2285

Hennekes H and Nigg EA (1994) The role of isoprenylation in membrane attachment of nuclear lamins. A single point mutation prevents proteolytic cleavage of the lamin A precursor and confers membrane binding properties. *J Cell Sci* **107**: 1019–1029

© Cancer Research Campaign 1997

Ito T, Kawata S, Tamura S, Igura T, Nagase T, Miyagawa J, Yamazaki E, Ishiguro H and Matsuzawa Y (1996) Suppression of human pancreatic cancer growth in BALB/c nude mice by manumycin, a farnesyl: protein transferase inhibitor. *Jpn J Cancer Res* **87**: 113–116

James GL, Goldstein JL, Brown MS, Rawson TE, Somers TC, McDowell RS and Crowley CW (1993) Benzodiazepine peptidomimetics: potent inhibitors of ras farnesylation in animal cells. *Science* **260**: 1937–1942

Kohl NE, Mosser SD, Jane Desolms S, Giuliani EA, Pompliano DL, Graham SL and Smith RL (1993) Selective inhibition of ras-dependent transformation by a farnesyltransferase inhibitor. *Science* **260**: 1934–1937

Krohne G, Waizenegger I and Höger TH (1989) The conserved carboxy-terminal cysteine of nuclear lamins is essential for lamin association with the nuclear envelope. *J Cell Biol* **109**: 2003–2011

Lowry OH, Rosebrough NJ, Farr AL and Randall RJ (1951) Protein measurement with the folin phenol reagent. *J Biol Chem* **193**: 265–275

Lowy DR and Willumsen BM (1993) Function and regulation of Ras. *Annu Rev Biochem* **62**: 851–891

Lutz RJ, Trujillo MA, Denham KS, Wenger L and Sinensky M (1992) Nucleoplasmic localization of prelamin A: implications for prenylation-dependent lamin A assembly into the nuclear lamina. *Proc Natl Acad Sci USA* **89**: 3000–3004

Maltese WA (1990) Posttranslational modification of proteins by isoprenoids in mammalian cells. *FASEB J* **4**: 3319–3328

Manne V, Roberts D, Tobin A, O'Rourke E, De Virgilio M, Meyers C, Ahmed N, Kurz B, Resh M, Kung H-F and Barbacid M (1990) Identification and preliminary characterization of protein-cysteine farnesyltransferase. *Proc Natl Acad Sci USA* **87**: 7541–7545

Nagase T, Kawata S, Tamura S, Matsuda Y, Inui Y, Yamasaki E, Ishiguro H, Ito T and Matsuzawa Y (1996) Inhibition of cell growth of human hepatoma cell line (Hep G2) by a farnesyl protein transferase inhibitor: a preferential suppression of ras farnesylation. *Int J Cancer* **65**: 620–626

Nagasu T, Yoshimatsu K, Rowell C, Lewis MD and Garcia AM (1995) Inhibition of human tumour xenograft growth by treatment with the farnesyl transferase inhibitor B956. *Cancer Res* **55**: 5310–5314

Omura S, Van Der Pyl D, Inokoshi J, Takahashi Y and Takeshima H (1993) Pepticinnamins, new farnesyl-protein transferase inhibitors produced by an actinomycete. I. Producing strain, fermentation, isolation and biological activity. *J Antibiot* **46**: 222–228

Reiss Y, Goldstein JL, Seabra MC, Casey PJ and Brown MS (1990) Inhibition of purified p21$^{ras}$ farnesyl: protein transferase by Cys-AAX tetrapeptides. *Cell* **62**: 81–88

Schaber MD, O'Hara MB, Garsky VM, Mosser SD, Bergstrom JD, Moores SL, Marshall MS, Friedman PA, Dixon RAF and Gibbs JB (1990) Polyisoprenylation of ras in vitro by a farnesyl-protein transferase. *J Biol Chem* **265**: 14701–14704

Sepp-Lorenzino L, Ma Z, Rands E, Kohl NE, Gibbs JB, Oliff A and Rosen N (1995) A peptidomimetic inhibitor of farnesyl: protein transferase blocks the anchorage-dependent and -independent growth of human tumor cell lines. *Cancer Res* **55**: 5302–5309

Sinensky M, Beck LA, Leonard S and Evans R (1990) Differential inhibitory effects of lovastatin on protein isoprenylation and sterol synthesis. *J Biol Chem* **265**: 19937–19941

Sinensky M, Trujillo MA and Lutz R (1991) Nuclear localization of prenylation dependent maturation of pre-lamin A (abstract). *FASEB J* **5**: 1181

Sinensky M, McLain TM and Fantle K (1994*a*) Expression of prelamin A but not mature lamin A confers sensitivity of DNA biosynthesis to lovastatin in F9 teratocarcinoma cells. *J Cell Sci* **107**: 2215–2218

Sinensky M, Fantle K, Trujillo MA, McLain TM, Kupfer A and Dalton M (1994*b*) The processing pathway of prelamin A. *J Cell Sci* **107**: 61–67

Stick R and Krohne G (1982) Immunological localization of the major architectural protein associated with the nuclear envelope of the *Xenopus laevis* oocyte. *Exp Cell Res* **138**: 319–330

Van Der Pyl D, Inokoshi J, Shiomi K, Yang H, Takeshima H and Omura S (1992) Inhibition of farnesyl-protein transferase by gliotoxin and acetylgliotoxin. *J Antibiot* **45**: 1802–1805

Wolda S and Glomset JA (1988) Evidence for modification of lamin B by a product of mevalonic acid. *J Biol Chem* **263**: 5997–6000

© Cancer Research Campaign 1997